# Understanding the Path Toward Financial Well-Being: Evidence From India

**DOI:** 10.3389/fpsyg.2021.638408

**Published:** 2021-07-21

**Authors:** Kanchan Sehrawat, Madhu Vij, Gaurav Talan

**Affiliations:** ^1^Faculty of Management Studies, University of Delhi, New Delhi, India; ^2^University School of Management Studies (USMS), Guru Gobind Singh Indraprastha University, New Delhi, India

**Keywords:** financial well-being, responsible financial behaviors, financial literacy, psychological factors, India

## Abstract

Many countries are taking steps to establish national strategies to improve the financial well-being (FWB) of their people. However, FWB as a term is still in the infancy stage with a handful of models developed in the context of developed countries. Thus, there is a need to understand FWB from a multi-disciplinary and multi-dimensional approach to draft and implement efficient strategies, especially in the context of developing countries like India. In this study, we have performed path analysis to identify the specific constituents of financial literacy, financial behavior (FinB), and personality traits that affect the FWB (perceived and objective) of an individual in Indian context. Survey responses of 349 respondents are analyzed to empirically validate the proposed relationships using the partial least squares structural equation modeling (PLS-SEM) approach. The analysis mostly provides support to existing literature and challenges some. The findings provide support to 12 out of 17 tested effects with eight hypotheses. The understanding of pathways that lead to increased FWB of individuals has the potential to facilitate effective policy-making and designing of curriculum to support efforts of individuals toward higher FWB and responsible FinBs.

## Introduction

Globally, individuals strive to improve their financial lives. They make financial decisions (spend, save, borrow, etc.) to grow their assets and protect their resources in pursuit of improving their financial status/well-being/health. However, financial decisions can prove to be particularly challenging. Individuals in today's world are witnessing a rapid change in the financial system because of a growing global economy, technological advancement, and proliferation in financial products and services (for instance easy availability of loans). Individuals can easily find themselves caught up in an unpropitious economic situation if it is not handled with a responsible financial behavior (FinB). Financial problems have the potential to negatively impact not only an individual but the economy at large. The World Bank (2013) notes that globally policymakers are concerned with how the financial well-being (FWB) of households can be improved to enhance the financial sector and increase its stability. It is essential to identify specific personality traits, knowledge, or behaviors that help some individuals to endure difficult times and flourish in good times as compared with others in similar situations. Such knowledge can be useful for various stakeholders to facilitate and coordinate their efforts to improve the FWB of individuals (Netemeyer et al., 2018; Riitsalu and Murakas, 2019). Despite the growing need to understand the antecedents of FWB, limited research has been conducted so far in this area (Collins and Urban, 2020). Holistic research on factors elucidating diversities in FWB is still in a nascent stage (Brüggen et al., 2017). Brüggen et al. (2017); Netemeyer et al. (2018); Collins and Urban (2020); and Riitsalu and Murakas (2019), among others, called for a more comprehensive and integrated approach to understand how FWB operates *vis-à-vis* various FinBs, knowledge, and personality traits.

The purpose of this research is thus 3-fold.

First, existing research in the field of personal finance is restricted to financial inclusion, financial literacy, capability, or specific FinBs. However, FWB is still a novel term in the financial inclusion community (Brüggen et al., 2017; Collins and Urban, 2020). Zyphur et al. (2015) pointed out that research in the area of FWB is still sparser than that of overall well-being. Existing studies either resort to objective measures, such as income, savings, debt-to-income ratio, or a single statement satisfaction question to gauge FWB. However, people in a similar objective financial condition may perceive their FWB differently (i.e., positively or negatively).

Consequently, individuals in an identical objectively measured financial situation may consider their personal FWB more or less positively (Grable et al., 2012). Therefore, the use of any single approach, i.e., subjective or objective, may not be suitable for evaluating a multifaceted and personal phenomenon such as FWB. To the best knowledge of the authors, no study has investigated the interplay between responsible FinBs, financial knowledge, psychological factors, and FWB (subjective and objective). This study aims to empirically test the said interplay in the Indian context.

Second, prior research mainly relies on objective financial knowledge and measurable behaviors while ignoring the role of confidence and motivation (Klapper et al., 2014; OECD, 2016). In contrast, according to their meta-analysis, Fernandes et al. (2014) asserted that financial literacy can predict a mere 0.1% of differences in FinBs. On the other hand, Vlaev and Elliott (2017) and Xiao and Porto (2017), among others, vouched for the higher role of confidence and motivation in explaining key FinBs. Knowledge of the interaction of FinBs with constituents of financial literacy can be instrumental in designing financial education programs to assist individuals in achieving their desired financial goals. This research considers the various constituents of financial literacy, i.e., objective knowledge, awareness, experience, and confidence levels, to throw light on the interplay of these components and their ability to predict FinBs and, ultimately, FWB.

Third, extant literature (Xiao and Dew, 2011; Kempson et al., 2013; Loke, 2017) tends to examine the effects of individual characteristics on one or more selected key behaviors separately. Incorporating various domains of personal financial management is essential since each of these domains has a different but significant impact on the well-being of individuals (Xiao and Dew, 2011). This study incorporates an array of FinBs to factor in various dimensions of personal financial management.

In this research, we aim to comprehensively understand the pathways to FWB and identify key predictors in the context of a developing economy, India. The results of this study can help understand the distribution of FWB across various sections of society, observe trends, and assess the effectiveness of prevalent policies, products, capabilities, and behavioral interventions.

## Conceptual Model

**Figure 1:**
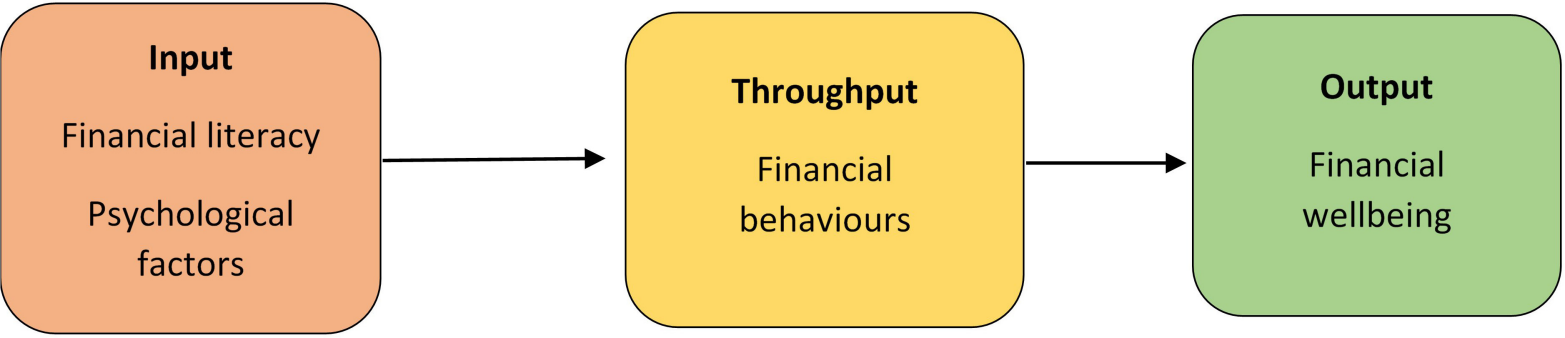
The conceptual framework adapted from Deacon and Firebaugh (1988). Source: Self depiction.

The conceptual model of this study finds its roots in the theory of family management system model as propounded by Deacon and Firebaugh (1988). In this study, a family resource management model is applied to individuals to analyze the components of their FWB, as depicted in Figure 1.

We conceptualize input or resources for throughput (i.e., responsible financial behavior, FinB) as components of financial literacy (i.e., awareness, objective financial knowledge, experience, and confidence) and psychological factors (i.e., time orientation, impulsivity, social status, self-control, and locus of control, LOC). The opinion of including psychological factors as input is supported by Mokhtar and Husniyah (2017). The throughput process has been measured using FinBs, namely, credit aversion, daily ease of meeting financial commitments, informed decision making, monitoring, informed product choices, spending attitudes, planning, and savings. The output component is characterized as FWB (objective and subjective).

**Figure 2:**
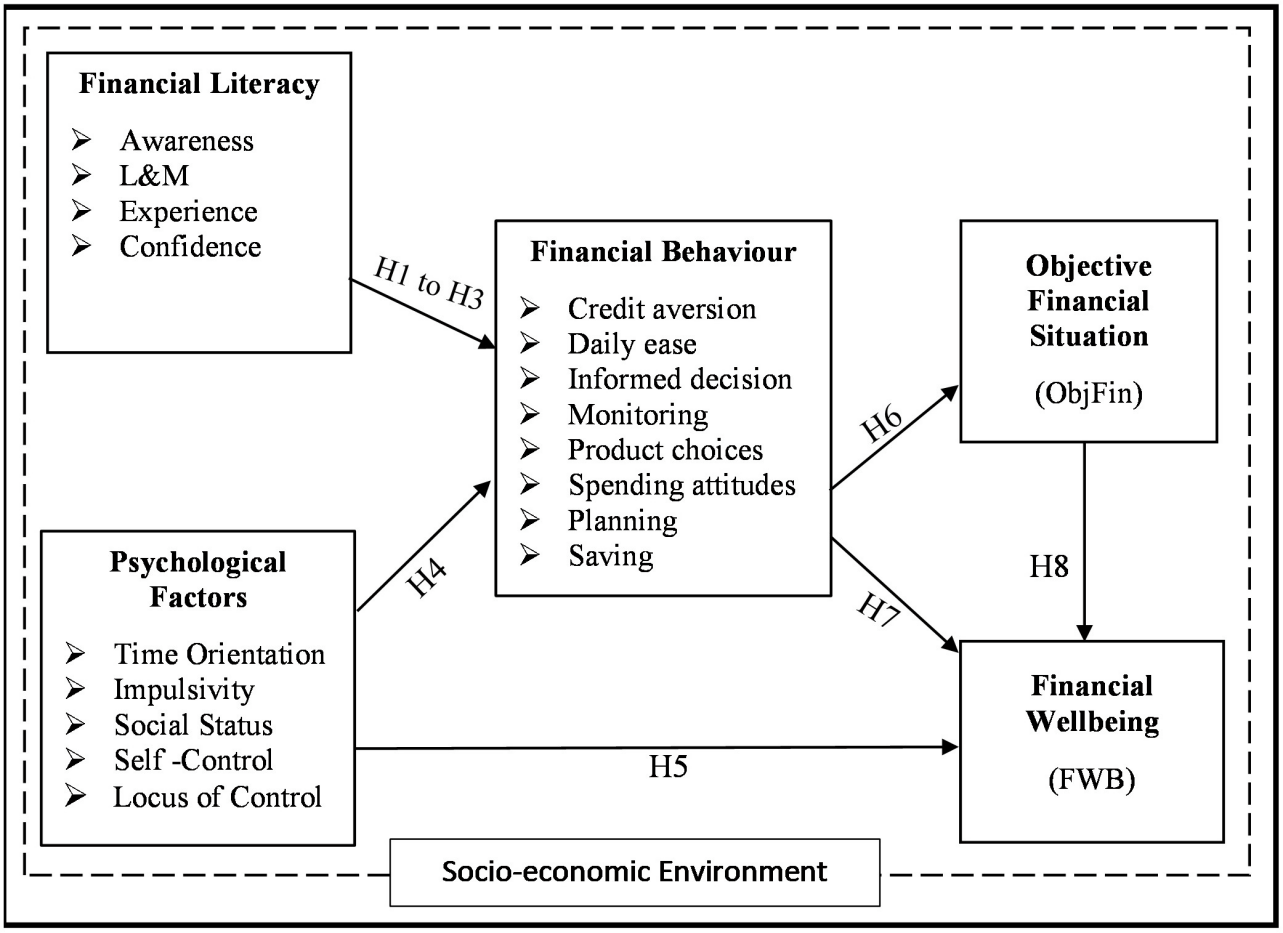
Conceptual Model. Source: Self depiction, L&M, Objective financial knowledge.

The elaborated conceptual model for this study, with additional interlinkage among the constructs, is illustrated in Figure 2. The model proposes that given a socio-economic environment, individuals with a high level of financial literacy are expected to have responsible FinBs, which are also influenced by the presence of positive psychological factors. Further, responsible FinBs are expected to affect FWB positively. This relationship can be either: (a) direct relationship, where particular FinB will affect perceptions of an individual of their FWB irrespective of how they are actually/objectively doing financially, or (b) indirect relationship, where particular FinBs will affect the objective financial situation, which would then possibly influence perceived FWB.

Socio-demographic characteristics are believed to shape the choices available to a person, how psychological characteristics predict behaviors, and how individuals perceive their well-being. Their effect is examined by correlational investigations unlike the relationships of other independent variables, which are explained as direct or indirect causal relationships. These relationships are analyzed by multigroup analysis (MGA) involving 440 comparisons of the proposed model.

The conceptual model shares many elements with the framework of the Consumer Financial Protection Bureau (CFPB) (2015) and Kempson et al. (2017). Both models conceptualized FWB to be primarily driven by FinBs, which, in turn, are ascertained by the resources of an individual. Further, both models consider knowledge, skills and attitudes, and psychological traits as indirect influencers of FWB through FinBs. In the view of the authors, psychological traits can also affect the perception of being financially well-directly. Further, the socio-economic environment is considered as a direct influencer for all the factors considered except behavior in Kempson et al. (2017), and behavior and well-being in CFPB (2015). In the view of the authors, the effect of socio-demographic variables should be considered in each relationship, i.e., they should be considered as moderators that can influence the strength and even the directions of the proposed relationships among all the constructs of the model. Lastly, unlike the two models, we also explicitly differentiate between the objective situation and the perception of well-being. This is an exploratory relationship proposed by the model to provide further insights into the existing knowledge of the pathways to FWB. The details of the constructs and the operational definitions used in the study are presented in Table 1.

**Table 1:** Operational definitions.

**Construct**	**Definition**	**Source**
Financial literacy	Combination of awareness, objective financial knowledge, and confidence those facilitate necessary responsible financial behavior which will ultimately help in achieving financial well-being	OECD (2013)
Responsible financial behaviors	Activities an individual undertake in terms of savings, investments, credit management, retirement planning, etc. to utilize the financial resources with the goal of economic satisfaction	Kempson et al. (2013)
Time orientation	It is the self-reported levels of consideration of future consequences of their actions	Rutledge and Deshpande (2015)
Impulsivity	It refers to action taken without giving much thought to its consequences	Fujita et al. (2006)
Self-control	It is the control an individual has on their actions in times of temptations	Baumeister (2002)
Locus of control	It is the level of control an individual feels they have over their behaviors and its consequences thereof	Rotter (1966)
Social status	It refers to the degreeto which an individualis concerned regarding the way other people view them or their status, as well astheir desire for people to respect them	Kempson and Poppe (2018)
Objective financial situation	It denotesthe objective realitiesof an individual's financial condition	CFPB (2018)
Financial well-being	“A state wherein a person can fully meet current and ongoing financial obligations can feel secure in their financial future, and can make choices that allow enjoyment of life”	CFPB (2015)

*Source: Own compilation*.

## Literature Review and Hypothesis Development

Managing personal finances and coping with financial exigency are essential for the well-being of an individual and their household, and the economy at large. As a response to this need, various stakeholders focus on financial inclusion, i.e., access to various financial products and services (Alliance for Financial Inclusion, 2016, 2017); financial literacy, i.e., building capacity (Lusardi and Mitchell, 2014); and financial capability, i.e., enhancement of knowledge and change in behavior (Stumm et al., 2013; CFPB, 2018). It can be gathered that the common aim of all the above mentioned is toward enhancing the overall FWB of an individual (Kempson and Poppe, 2018). However, FWB is still a novel concept that lacks a conceptual definition and standardized measurement scales.

A handful of studies have attempted to explore comprehensive FWB models. Further, existing proposed models of FWB are predominantly established and examined in developed countries, such as the United States (CFPB, 2015), Norway (Kempson et al., 2017), the United Kingdom (Hayes et al., 2016), Canada (Financial Consumer Agency of Canada, 2018), Australia, and New Zealand (Prendergast et al., 2018a,b). It is important to understand that the theories developed and evidence obtained from data of developed economies may not apply in developing economies (Burgess and Steenkamp, 2006). Xiao et al. (2014) asserted that it is necessary to carry out studies in other developed and developing economies to enhance the understanding of FWB in various socio-economic contexts. Indian studies have largely focused on financial inclusion and financial literacy. However, only few research studies have ventured into the novel concept of FWB.

The gap relating to personal finance and FWB literature could be summarized in the necessity of proposing a holistic model, which can accurately identify the pathways to the FWB of Indians. Hence, in order to fill this gap, this study aims to empirically examine the relationship of responsible FinBs with FWB and how these relationships are determined by various psychological factors, financial literacy components, and sociodemographic factors.

### Relationship Between Financial Literacy and Responsible Financial Behavior

Literature highlights a positive relationship between financial literacy and FinBs (Allgood and Walstad, 2016; Bannier and Neubert, 2016). The financial outcome that is part of the study is FWB, which is mainly reported to have a positive correlation with financial literacy (Addin et al., 2013; Sabri and Zakaria, 2015). However, no substantial relationship between financial literacy and FWB is documented by (Shim et al., 2009). Further, Courchane and Zorn (2005) reported a sequential effect of financial knowledge, FinB, and financial performance.

Lee and Hanna (2014) reported that financial knowledge affects the attitude an individual has toward awareness about money management. This attitude affects the actual money management behavior, which, in turn, affects the outcomes of such money management. Financial knowledge, along with financial decision-making experience, has a potential to reduce the likelihood of individuals to get trapped in unscrupulous sales practices of financial product and service providers (Atia, 2012). Lyons (2008) and Rao and Barber (2005) reported that financial education influences FWB through FinBs.

Thus, it can be inferred that an individual who is aware of available financial products and services is expected to have the higher objective financial knowledge and higher financial confidence. This further leads to higher experience in financial decision-making, which ultimately influences FinBs. We, therefore, hypothesized that components of financial literacy have a positive relationship with responsible FinBs.

*H1: There is a positive and significant association between financial awareness and FinB via objective financial knowledge (L and M) and financial experience (FinExp)*.

*H2*_*a*_*: There is a positive and significant association between financial awareness and FinB via financial confidence*.

*H2*_*b*_*: There is a positive and significant association between financial awareness and FinB via financial confidence and financial experience*.

*H3: There is a positive and significant association between objective financial knowledge (L and M) and FinB via FinExp*.

### Relationship Between Psychological Factors, Responsible Financial Behaviors, and Financial Well-Being

Literature suggests several potential psychological traits that influence financial decision making and FWB. The psychological factors that are incorporated into the conceptual model of this study are described briefly in subsequent subsections.

### Time Orientation

Time orientation can be comprehended as the preference of immediate or current gratification over possible future gains. Short-term time orientation (TO), i.e., urge of immediate gratification, is identified as a key inhibitor of financial effectiveness (Vyvyan et al., 2014). In fact, Shepard and Turner (2019) provided evidence that the positive relationship of future orientation with well-being (measured in terms of health, happiness, life satisfaction, and FWB) is robust across cultures and countries in their sample of 64 countries. Kooij et al. (2018), in their meta-analysis, concluded that having future time perspectives are subjective expectations and beliefs of an individual about their future, which include the ability to set and pursue long-term goals as well as to delay gratification. Further, they concluded that it is a crucial factor that fosters health and well-being.

Literature provides evidence that TO influences several financial contexts, such as retirement savings (Hastings et al., 2011), credit (Benton et al., 2007), risk tolerance (Jacobs-Lawson and Hershey, 2005), savings (Kempson and Poppe, 2018), financial planning (Hershey et al., 2010), compulsive buying or spending restraint (Kempson and Poppe, 2018) and financial or economic well-being (Kooij et al., 2018; Shepard and Turner, 2019).

It can, thus, be anticipated that short-term TO is negatively related to responsible FinB and FWB.

*H4*_*a*_*: There is a negative and significant association between short-term TO and responsible FinBs*.

*H5*_*a*_*: There is a negative and significant association between short-term TO and perceived FWB*.

### Impulsivity (Impulse)

Impulsivity encompasses a trade-off between long term benefits and immediate satisfaction (Vohs et al., 2012; Bernheim et al., 2015). In terms of FinB, impulsive people seek immediate benefits and make short term financial decisions instead of making decisions that are consistent with their long-term financial goals. Fujita et al. (2006) provide evidence that individuals with high impulsivity tend to act in a non-optimal way. Impulsivity is negatively related to FWB (Kempson and Poppe, 2018). Impulsivity is linked to several negative FinBs like over-indebtedness (Abrantes-Braga and Veludo-de-Oliveira, 2020), financial instability (Lusardi et al., 2010), holding low-interest saving products (Gathergood and Weber, 2014), and low retirement savings (DeHart et al., 2016). It can thus be anticipated that high impulsivity is negatively related to responsible FinB and FWB.

*H4*_*b*_*: There is a negative and significant association between high impulsivity (impulse) and responsible FinB*.

*H5*_*b*_*: There is a negative and significant association between high impulsivity and perceived FWB*.

### Self-Control

Self-control denotes the capability of controlling the temptation or controlling one's impulses, emotions, actions, and desires to protect a valued goal (Gerhard et al., 2018). Self-control (SC) has been reported as an important predictor of success in several domains of life, such as better educational attainment (Duckworth and Seligman, 2005), and better FinB and FWB (Strömbäck et al., 2017). However, based on their experimental study, Ballinger et al. (2011) argued that the relationship is yet to be established between SC and responsible FinB, such as saving.

Self-control is linked with specific positive financial behaviors, such as spending and borrowing restraint (Achtziger et al., 2015; Kempson and Poppe, 2018), retirement planning, and saving (Strömbäck et al., 2017), asset diversification and wealth (Biljanovska and Palligkinis, 2018), credit score (Arya et al., 2013), and positive FinB in general (Miotto and Parente, 2015).

Though the two constructs, self-control and impulsivity, are inter-related, they are believed to stem from different neurological bases (Lieberman, 2007; Steinberg, 2008) and, thus, it is crucial to consider both, especially in the case of evaluating a decision-making process (Chen and Vazsonyi, 2011).

From the above discussions, it can be anticipated that high SC can lead to responsible FinB and FWB.

*H4*_*c*_*: There is a positive and significant association between high SC and responsible FinBs*.

*H5*_*c*_*: There is a positive and significant association between high SC and perceived FWB*.

### Locus of Control

Locus of control is the extent to which one feels in control of events that affect them (Hellrigel et al., 2010). Individual's perceived control over outcomes have a significant impact on their financial prosperity (Perry and Morris, 2005). LOC is an important intra-personal component of empowerment which is a strong influencer (both direct and indirect) on financial habits (Angulo-Ruiz and Pergelova, 2015).

Individuals with an internal LOC will demonstrate responsible financial management behavior, as evident in the studies of Angulo-Ruiz and Pergelova (2015) and Mien and Thao (2015), and higher FWB (Prawitz and Cohart, 2016; Kempson and Poppe, 2018; Mahdzan et al., 2019). They also have higher savings and consumption rates (Cobb-Clark et al., 2016; Kempson and Poppe, 2018), better investment returns (Salamanca et al., 2020), low dependence on welfare receipt (Chan, 2017), higher wealth accumulation (Cobb-Clark et al., 2016); and they make informed product choice (Hoffman et al., 2003). It can, thus, be anticipated that higher LOC is positively related to responsible FinB and FWB.

*H4*_*d*_*: There is a positive and significant relationship between internal LOC and responsible FinB*.

*H5*_*d*_*: There is a positive and significant association between internal LOC and perceived FWB*.

### Social Status

Social status can be understood as the inclination of individuals to follow social norms, also referred to as social validation, herding, or social proof. The process of social comparisons plays an important role in the perception of own financial situation (Clark and Senik, 2010). The perception of financial status in comparison to one's peer group is often a stronger predictor for different behaviors than objective measures. This “keeping up with the Joneses” effect is likely to influence monetary behavior (Masche, 2010), participation in employer-sponsored retirement plans (Duflo and Saez, 2002), stock market participation (Sivaramakrishnan et al., 2017), purchase decisions (Attri, 2013), higher savings (Raue et al., 2020), and prevent incapable borrowing (Kempson and Poppe, 2018).

Thus, it can be inferred that comparing oneself with others tends to promote goal attainment and provides motivation to engage in positive FinBs (Frederiks et al., 2015). Similarly, Kempson et al. (2013) provided evidence of a positive relationship of social status with financial capabilities. Sundarasen et al. (2016) reported a positive influence of social comparison on financial planning and practices. In contrast, Rahman and Gan (2020) revealed no relationship between herding behavior and investment decisions. Furthermore, Money Advice Service (2015) argued that social norms strongly but negatively influence FinBs. Thus, social status, where an individual tries to fit into the social group, can act as a facilitator or barrier to responsible FinB. Norvilitis and Mendes-Da-Silva (2013) and Norvilitis and Mao (2013) revealed a negative relationship between social comparisons and FWB among college students. A positive but low relation is reported by Prendergast et al. (2018a), whereas a non-significant relation is observed by Prendergast et al. (2018b). A negative relationship of social status with subjective FWB is evident in the study of Kempson and Poppe (2018).

It can, thus, be anticipated that high concern for social status is positively related to responsible FinB. However, it holds a negative relation with subjective FWB.

*H4*_*e*_*: There is a positive association between high concern for social status (SS) and responsible FinBs*.

*H5*_*e*_*: There is a negative and significant association between high concern for SS and perceived FWB*.

### Relationship Between Responsible Financial Behaviors and Financial Well-Being

This study conceptualizes responsible FinB as having eight components, namely, credit aversion, daily ease of meeting financial commitments, informed decision making, monitoring financial activities, product choices, spending attitudes, planning, saving, and investments.

Credit usage has been linked to several problems, such as psychological distress, lower self-esteem, depression, humiliation, and anxiety, which have an adversarial effect on physical and mental health (Hojman et al., 2016). It is also associated with reduced perceived FWB (Norvilitis et al., 2003). The problem in meeting daily financial commitments with ease may result in the use of credit for meeting these “day-to-day” commitments, which can adversely affect the FWB of an individual (Delafrooz and Paim, 2013; Finney, 2016). In the model, we have, thus, included credit aversion and ease of meeting daily commitments in the basket of responsible FinBs.

Making budgets and regular comparisons of actual and planned expenditures can help individuals with their routine money management, which is positively associated with their well-being (Sabri and Zakaria, 2015). Individuals involved in making informed decisions by exploring product options and regularly monitoring their expenses against their incomes are expected to have higher FWB (Kempson et al., 2013). High propensity to spend money on non-essential items has an inverse relation with FWB (Delafrooz and Paim, 2013). Stress due to unhealthy spending can also reduce physical health and results in lower job performance (Dunn and Mirzaie, 2012). In the model, we measured spending restraint, which is the ability not to indulge in overspending and, thus, is proposed to have a positive impact on FWB.

A healthy balance between spending and savings is imperative for sustaining FWB in the long run (Van Praag et al., 2003). Higher propensity to save is related to improved barraging power or decision-making authority (Schaner, 2017), lower possibilities of selling assets to meet financial emergencies (Jack and Suri, 2014), increased productivity (Knowles, 2013), and ultimately better FWB (Kempson et al., 2013).

Knowledge of financial behaviors in which individuals are involved allows them to deliberate more judiciously about what is necessary to enhance their future financial prospects. Thus, we can propose that an array of responsible FinBs, future-oriented behaviors one was previously engaged in, will be positively related to perceived FWB. As also asserted by Huston (2010), Netemeyer et al. (2018), and Perry and Morris (2005) in their respective studies, FWB is the outcome of FinBs.

Further, after reviewing the published literature and drawing from their conceptual frameworks. It was revealed that FWB consists of two primary components.

- Financial consequences that individuals encounter and testify through their personal subjective lens, and
- Financial consequences that are observable from financial records, accounts, and transactions of an individual.

This study relies on the self-reported subjective or perceived FWB and derived a score of objective financial situation. The exploratory links of FinB with objective financial situation and that with perceived FWB are hypothesized to have positive relations. This implies that individuals who engage in responsible FinB are expected to experience higher FWB (both perceived and actual). It is further proposed that achieving higher objectively measured FWB by engaging in responsible FinB further enhances the perceptions of being financially well.

*H6: There is a positive and significant association between responsible FinB and objective financial situation (ObjFin)*.

*H7: There is a positive and significant association between responsible FinB and perceived FWB*.

*H8: There is a positive and significant association between ObjFin and perceived FWB*.

## Materials and Methods

### Sample Size and Data Collection

This study utilized a structured questionnaire to gather data for hypothesis testing and to address the research objectives. The overall population of the study includes any individual who is either aged (i) 24 years or above or (ii) 18 years and above with a work experience of more than 2 years. In general, there is no consensus on how to calculate the sample size for PLS-SEM. Various rules of thumb and software are at disposal of the researchers. Hair et al. (2006) recommended a minimum of 200 respondents as sample size. Further, Brysbaert (2019) argued that running more participants than strictly needed involves a minor financial cost, whereas running fewer participants entails an increased risk of drawing incorrect conclusions. Thus, we aimed to get a sample size higher than 200 to draw meaningful conclusions in light of published literature. A useable sample of 349 responses was achieved with time and financial constraints.

Data for this study was collected by offline (pen and paper) as well as an online survey. The invitation link to the survey site (surveymonkey.com) was sent through emails, and social networking platforms, such as WhatsApp, LinkedIn, and Facebook, between July 2019 and October 2019. A total of 269 responses were gathered utilizing the survey platform, and the in-person survey received 225 questionnaires. Out of the total 494 responses, 394 were found to be complete and met the criteria of the study.

Table 2 explains the profile of the 394 respondents, and among them, 58% were male and 42% were female. An approximately equal number of respondents reported their marital status to be either married or single, and a low number of respondents with divorce or separated status could be reached. Only 4% respondents have educational qualifications less than a gradation or diploma. Around 60% of the respondents do not have any dependent adult or child in the sample. A diverse occupation respondent base is achieved in the sample, with most of the respondents working in the private sector.

**Table 2:** Demographic profile of respondents.

**Gender**			**Marital status**		
**Particulars**	***n***	**%**	**Particulars**	***n***	**%**
Male	228	57.9	Single i.e., never married	189	48.0
Female	166	42.1	Married	197	50.0
Total	394	100.0	Separated/Divorced	8	2.0
			Total	394	100.0
**Age groups**			**Work experience**		
18–24 years	75	19.0	Never worked	16	4.1
25–34 years	199	50.5	<1 year	14	3.6
35–44 years	62	15.7	1–5 years	172	43.7
45–54 years	41	10.4	5–10 years	83	21.1
55–60 years	11	2.8	10–15 years	34	8.6
61 years & above	6	1.5	More than 15 years	75	19.0
Total	394	100.0	Total	394	100.0
**Dependent adult**			**Dependent child**		
No dependent	228	57.9	No child	244	61.9
Dependent adult	166	42.1	Dependent child	150	38.1
Total	394	100.0	Total	394	100.0
**Occupation**			**Monthly income**		
Govt/PSU	116	29.4	Below 15,000	33	8.4
Homemaker	15	3.8	15,000–24,999	52	13.2
Owns a business	29	7.4	25,000–34,999	53	13.5
Private sector	197	50.0	35,000–44,999	40	10.2
Retired	5	1.3	45,000–54,999	43	10.9
Self-Employed	17	4.3	55,000–64,999	33	8.4
Student	10	2.5	65,000–74,999	28	7.1
Not employed	5	1.3	75,000–100,000	45	11.4
Total	394	100.0	Above 100,000	67	17.0
			Total	394	100.0
			**Education**		
			Higher secondary	14	3.55
			Graduation/Diploma	191	48.5
			PG/PD or above	189	48.0
			Total	394	100.0

*Source: Survey results. PG, post-graduation; PD, professional degree*.

### Instrument Development

Literature review helped in generating the items on a provisional basis for inclusion in the questionnaire. The construct items were refined with the help of expert interviews. The purification included rewording of the items, editing, adding, and deleting and revising the item statements. This step was followed by face validity and content validity. Subsequently, all the scales were put together in the form of a questionnaire and underwent a pilot test on a convenience sample of 50 respondents.

The final questionnaire is divided into five sections. The first section aims to collect general information and includes the filter questions. The second section deals with day-to-day or month-to-month money management. The third section seeks to collect information regarding savings and investment patterns, followed by the fourth section that records the responses on various risk and risk mitigation strategies followed by the respondent. The last section assesses financial capabilities in terms of decision-making and financial knowledge.

Perceived financial well-being is measured using the original five-item scale developed by CFPB (2017). Objective FWB is evaluated based on seven items, namely, difficulty in making ends meet (ObjFin_1), savings levels (ObjFin_2), ability to absorb negative shock (ObjFin_3), diversification of investment portfolio (ObjFin_4), unpaid loans (ObjFin_5), retirement planning (ObjFin_6), and insurance plan (ObjFin_7).

Psychological factors are measured using a three-item scale on a five-point Likert scale for each of the construct, namely, TO, impulsivity (impulse), SS, SC, and, LOC. The scales are adapted from Antonides et al. (2011), Kempson et al. (2013), Kempson et al. (2017), Perry and Morris (2005), and Prendergast et al. (2018a,b). Responsible FinB in terms of spending restraint is measured using three items adapted from Kempson et al. (2017) and Prendergast et al. (2018a,b). Behavior pertaining to monitoring personal finance, informed decision-making, and product choice is measured using items adapted from Prendergast et al. (2018a,b). Personal financial planning behavior is measured using two items similar to the study of Hayes et al. (2016). Credit aversion behavior is measured on three statements adapted from the FinScope Survey (2017). Active saving behavior is measured using three statements as in Kempson et al. (2013) and Prendergast et al. (2018a,b).

Financial confidence questions are adapted from the study of Farrell et al. (2016), and financial experience (FinEx) is measured with adapted items from the studies of Comerton-Forde et al. (2018) and OECD (2016). Financial awareness is measured by preparing a 20-item list relevant in Indian context grounded on the description of financial literacy (OECD, 2005). Objective financial literacy is evaluated using the five questions developed by Lusardi and Mitchell (2011), also known as the “Big5” questions.

### Measures

We have measured psychological factors and subjective FWB as first-order reflective constructs, whereas financial literacy and objective FWB are a first-order formative construct. The formative construct assumes that its indicators cause the construct i.e., the selected indicators encompass all the vital aspects of the particular domain. On the other hand, reflective indicators are produced by the construct i.e., the indicators are highly correlated, and any deletion of an indicator does not change the meaning of the latent variable. Responsible FinB is theorized as a higher-order reflective-formative construct. The multiple reflective indicators are adapted from the extant literature. The formative indicators are adapted from literature and purified with the help of expert interviews. These indicators are then combined and modified to fit the context of this research.

Based on Hair et al. (2017) recommendations, we performed partial least squares modeling (PLS-SEM) (SmartPLS 3.2.6) to access the inter-relationships of various constructs, as proposed in the conceptual model. PLS-SEM is a second-generation advanced statistical technique. It incorporates the characteristics of factor analysis and multiple regression, which facilitate the simultaneous examination of direct and indirect effects of exogeneous and endogenous variables. Thus, PLS-SEM enables working on complex models. To test for statistical significance, we resorted to bootstrapping with 5,000 re-samplings.

## Data Analysis and Results

On preliminary analysis of the total 494 responses, missing or invalid responses were observed in 100 survey responses, thus leaving 394 responses for the final empirical analysis. The normality is gauged by comparing the skewness and kurtosis of all the interval scale data. The indicators are under the acceptable value of 2 (skewness) with two exceptions, i.e., credit aversion 3 (−2.164) and ObjFin_1 (−2.649). Further, none of the kurtosis values of an indicator exceeds 7, indicating no significant issues with univariate normality. Following the preliminary analysis, we moved toward the evaluation of measurement and structural models.

It is also important to note here that in the proposed model construct “FinB,” responsible financial behavior, is a reflective-formative type hierarchical component model. To obtain the true relationships of the latent variables on higher order constructs (HOC) in the structural path model, the two-stage approach is employed as suggested by Henseler and Chin (2010). In stage one, a repeated indicator approach is used to obtain the latent variable scores for lower-order constructs of “FinB;” and in stage two, these latent variable scores are used for computing the full model. Further, the assessment of the HOC is in line with the procedure followed for other constructs.

### Evaluation of Measurement Model

#### Reflective Measurement Model

The summary of reflective measurement model evaluation is presented in Table 3.

**Table 3:** Summary of test results for reflective measurement model.

**LV**	**Indicators**	**Indicator reliability**	**Internal consistency**		**Conv. validity**	**Discriminant validity**
		**Loadings**	**α**	**CR**	**AVE**	
Monitor	Monitor1	0.854	0.543	0.778	0.638	Yes
	Monitor2	0.740				
Plan	Plan1	0.921	0.826	0.920	0.852	Yes
	Plan2	0.924				
Spend	Spend1	0.736	0.712	0.838	0.634	Yes
	Spend2	0.767				
	Spend3	0.878				
Save	activesave1	0.830	0.757	0.861	0.673	Yes
	activesave2	0.853				
	activesave3	0.777				
Credit	credit_averse1	0.799	0.723	0.841	0.639	Yes
	credit_averse2	0.848				
	credit_averse3	0.748				
InformD	informed_decision1	0.808	0.565	0.821	0.696	Yes
	informed_decision2	0.860				
PC	productchoice1	0.910	0.837	0.903	0.756	Yes
	productchoice2	0.908				
	productchoice3	0.785				
DE	Dailyease		Single item construct			
FWB	FWB1	0.746	0.708	0.811	0.467	Yes
	FWB2	0.745				
	FWB3	0.776				
	FWB4	0.597				
	FWB5	0.514				
Impulse	Impulse1	0.874	0.654	0.800	0.581	Yes
	Impulse2	0.834				
	Impulse3	0.533				
LOC	LOC1	0.643	0.558	0.739	0.494	Yes
	LOC2	0.555				
	LOC3	0.923				
SC	SC1	0.740	0.615	0.794	0.563	Yes
	SC2	0.773				
	SC3	0.738				
SS	SS1	0.895	0.740	0.843	0.643	Yes
	SS2	0.801				
	SS3	0.698				
TO	TO1	0.878	0.611	0.789	0.563	Yes
	TO2	0.779				
	TO3	0.558				

*Source: Self depiction. α, Cronbach's alpha; CR, composite reliability; conv. validity, convergent validity*.

The internal consistency is gauged using Cronbach's alpha and composite reliability (CR). The Cronbach's alpha of all the constructs, except monitor, informD, and LOC, meet the generally accepted limit of. 6–0.7 (Hair et al., 2006). Further, each of the latent variables has acceptable CR and ranges from 0.778 (Monitor) to 0.92 (Plan) for our reflective scales (Hair et al., 2017). As Cronbach's alpha is sensitive to the number of items in the construct, and it works on a very strong assumption of equal reliability of all the indicators. Moreover, PLS-SEM prioritizes the indicators based on their individual reliability. We, thus, retain all the constructs, as their reliability is established by a CR test. Thus, we can conclude that all the indicators show high internal consistency.

Indicator reliability is established using outer loadings. The factor loadings should be higher than 0.7. However, indicators with outer loadings between 0.4 and 0.7 can be taken into account if the other criteria are fulfilled (Hair et al., 2017). The factor loadings in the model range from 0.514 (FWB5) to 0.924 (FinB_Plan2), satisfying the psychometric reliability test requirements (Henseler et al., 2009).

Convergent validity is established using average variance explained (AVE) criteria, and except for latent variables FWB and LOC, the AVE for each of the indicator is above 0.5, which signifies that the constructs can capture more than 50% of the variation in relation to the variance due to measurement error. However, FWB is short of only 0.033 and LOC of 0.006 from the required minimum value. Since these differences are very low, we retain the two constructs in their original form.

Discriminant validity is established using Fornell–Larcker, cross loadings, and HTMT (Hair et al., 2017), confirming that there are no discriminant validity issues in the model both at construct and item levels analyzed using the three criteria (refer to Appendix A for result tables).

#### Formative Measurement Model

Construct validity is established using discriminant validity. Discriminant validity among all the constructs is established using the Fornell–Larcker criterion (Fornell and Larcker, 1981) and the HTMT criterion (Henseler et al., 2015), indicating no discriminant validity issues (refer to Appendix B).

Indicator validity is gauged using variance inflation factor (VIF) and indicator weights. Each formative indicator is unique, and any change in these indicators may change the meaning of the entire construct. Hence, the correlation among the formative indicators is not desirable and expected. To gauge the collinearity among the indicators, VIF is analyzed. A lower value is acceptable, as it highlights lower levels of inflated variance. Table 4 presents the VIF values of all the formative constructs of the model, which are below three, indicating no collinearity issue in the data (Becker et al., 2015).

**Table 4:** Collinearity assessment.

**Latent Variable**	**VIF**	**Latent Variable**	**VIF**
**Formative construct: FinB**			
FinB_credit_averse1	1.621	FinB_productchoice1	2.957
FinB_credit_averse2	1.464	FinB_productchoice2	2.952
FinB_credit_averse3	1.320	FinB_productchoice3	1.489
FinB_Mointor1	1.086	FinB_Plan1	1.978
FinB_Mointor2	1.086	FinB_Plan2	1.978
FinB_Spend1	1.354	FinB_activesave1	1.691
FinB_Spend2	1.366	FinB_activesave2	1.732
FinB_Spend3	1.583	FinB_activesave3	1.350
FinB_informed_decision1	1.412	FinB_informed_decision2	1.552
**Formative construct: ObjFin**			
ObjFin_1	1.029	ObjFin_5	1.041
ObjFin_2	1.119	ObjFin_6	1.105
ObjFin_3	1.177	ObjFin_7	1.162
ObjFin_4	1.249		

*Source: Survey results*.

Table 5 shows that the weights of all the indicators of the formative construct financial behavior (FinB) are higher than 0.1 and significant on applying bootstrapping procedure indicating indicator validity (Hair et al., 2017).

**Table 5:** Significance and relevance of formative construct FinB.

**Formative construct**	**Latent variable**	**Outerweight**	***t*-statistics**	***p*-values**
FinB	Credit	0.238	30.201^***^	0.000
	DE	0.100	22.546^***^	0.000
	InformD	0.172	25.198^***^	0.000
	Monitor	0.158	27.829^***^	0.000
	PC	0.253	32.316^***^	0.000
	Plan	0.178	29.675^***^	0.000
	Spend	0.236	31.256^***^	0.000
	Save	0.244	33.657^***^	0.000

*Source: PLS-SEM output;*

*** *p <0.01 at 5,000 bootstraps*.

At the first iteration, the two indicators, ObjFin_5 (*t* = 1.309; *p* = 0.191) and ObjFin_7 (*t* = 1.076 and *p* = 0.282), of the formative construct objective financial situation (ObjFin) are not found to be significant. Adhering to the recommendation of Hair et al. (2017), the outer loading of formative indicators with non-significant weight is checked to gauge their absolute contribution to the indicator ObjFin. The outer-loading of ObjFin_5 is 0.035 and that of ObJFin_7 is 0.182; and as both the outer loadings are below the recommended level of 0.5, the indicators fail to be relatively and absolutely important when tested empirically. Thus, indicator Objfin_7 that represents the insurance ownership is dropped from the construct. However, during the content validation stage, experts have marked the status of unpaid loans measured by ObjFin5 as very important to determine the objective financial situation of the individual. Further, established literature supports a significant negative relation of unpaid loans with the overall FWB of the individual (Hojman et al., 2016; Blomgren et al., 2017). Hence the indicator is retained in the final construct (Table 6).

**Table 6:** Significance and relevance of formative construct ObjFin iteration 2.

**Formative construct**	**Indicator**	**Weights**	***t*-statistic**	***p*-value**
ObjFin	ObjFin_1	0.598	6.333^***^	0.000
	ObjFin_2	0.308	2.976^***^	0.003
	ObjFin_3	0.322	3.333^***^	0.001
	ObjFin_4	0.354	3.759^***^	0.000
	ObjFin_5	0.121	1.267	0.205
	ObjFin_6	0.188	1.898^*^	0.058

*Source: SmartPLS*.

*** *p <0.01*;

* *p <0.1 at 5,000 bootstraps*.

### Evaluation of Structural Model

#### Collinearity Assessment

Table 7 reports that all the VIF values of the construct indicators are below 2, which specifies that variances of regression coefficient estimator, i.e., *Var*(*b*_*i*_) are not inflated beyond the recommended limits. Thus, uniqueness of each construct indicator is established, indicating that there is no multi-collinearity issue in the structural model (Lowry and Gaskin, 2014).

**Table 7:** Collinearity assessment.

**Indicator**	**VIF**	**Indicator**	**VIF**	**Indicator**	**VIF**
ObjFin_1	1.023	LOC1	1.245	Impulse1	1.371
ObjFin_2	1.118	LOC2	1.224	Impulse2	1.495
ObjFin_3	1.162	LOC3	1.089	Impulse3	1.179
ObjFin_4	1.128	SC1	1.253	SS1	1.477
ObjFin_5	1.038	SC2	1.187	SS2	1.683
ObjFin_6	1.105	SC3	1.226	SS3	1.39
FWB1	1.542	FC_Aware_Total	1	TO1	1.39
FWB2	1.505	FC_ExpTscore	1	TO2	1.407
FWB3	1.564	FC_confidenceT	1	TO3	1.096
FWB4	1.353	LM_Total	1		
FWB5	1.067	LV_FinB	1		

*Source: SmartPLS output*.

#### Significance and Relevance of the Structural Model

**Figure 3:**
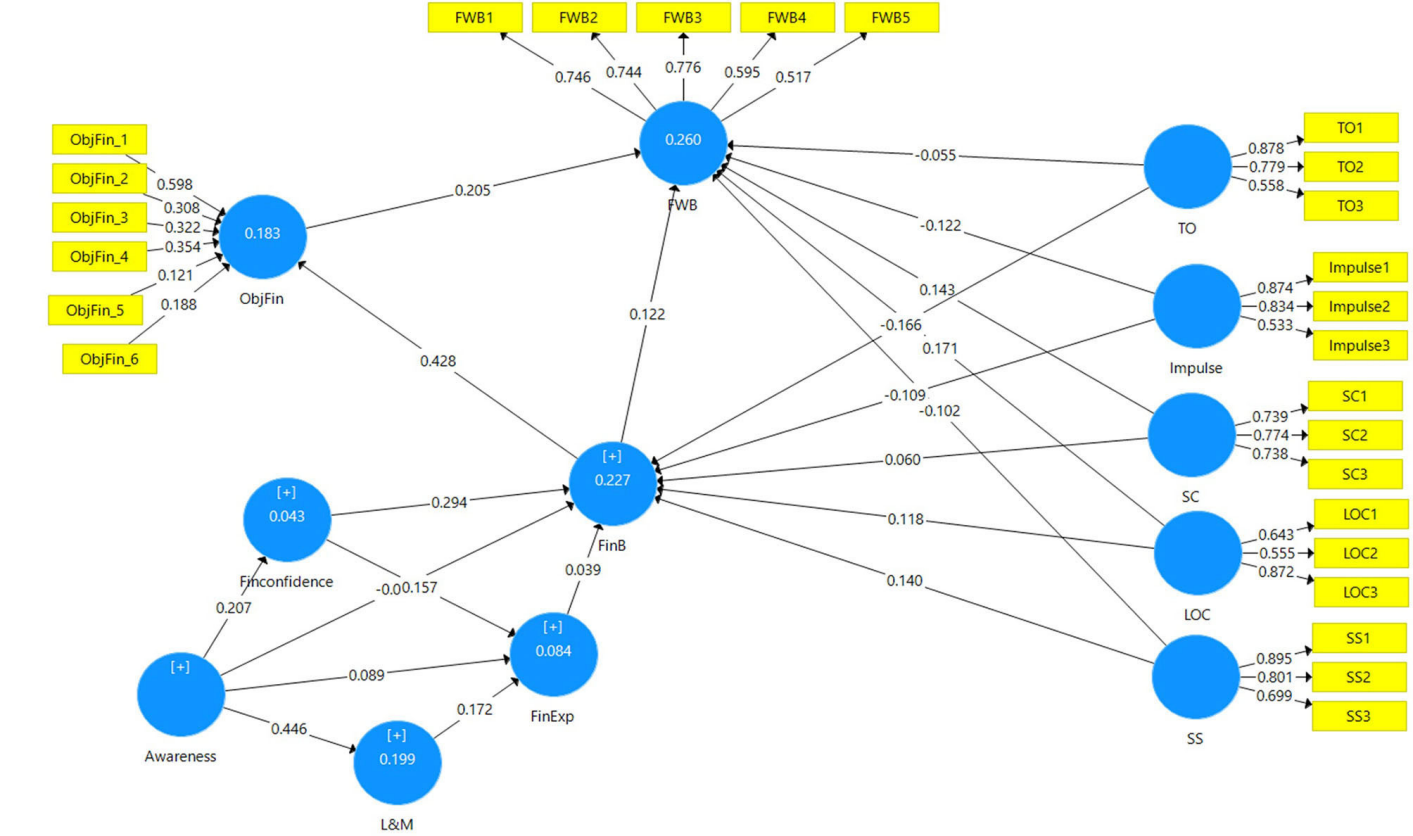
Significance of the structural model paths. Source: SmartPLS output.

**Figure 4:**
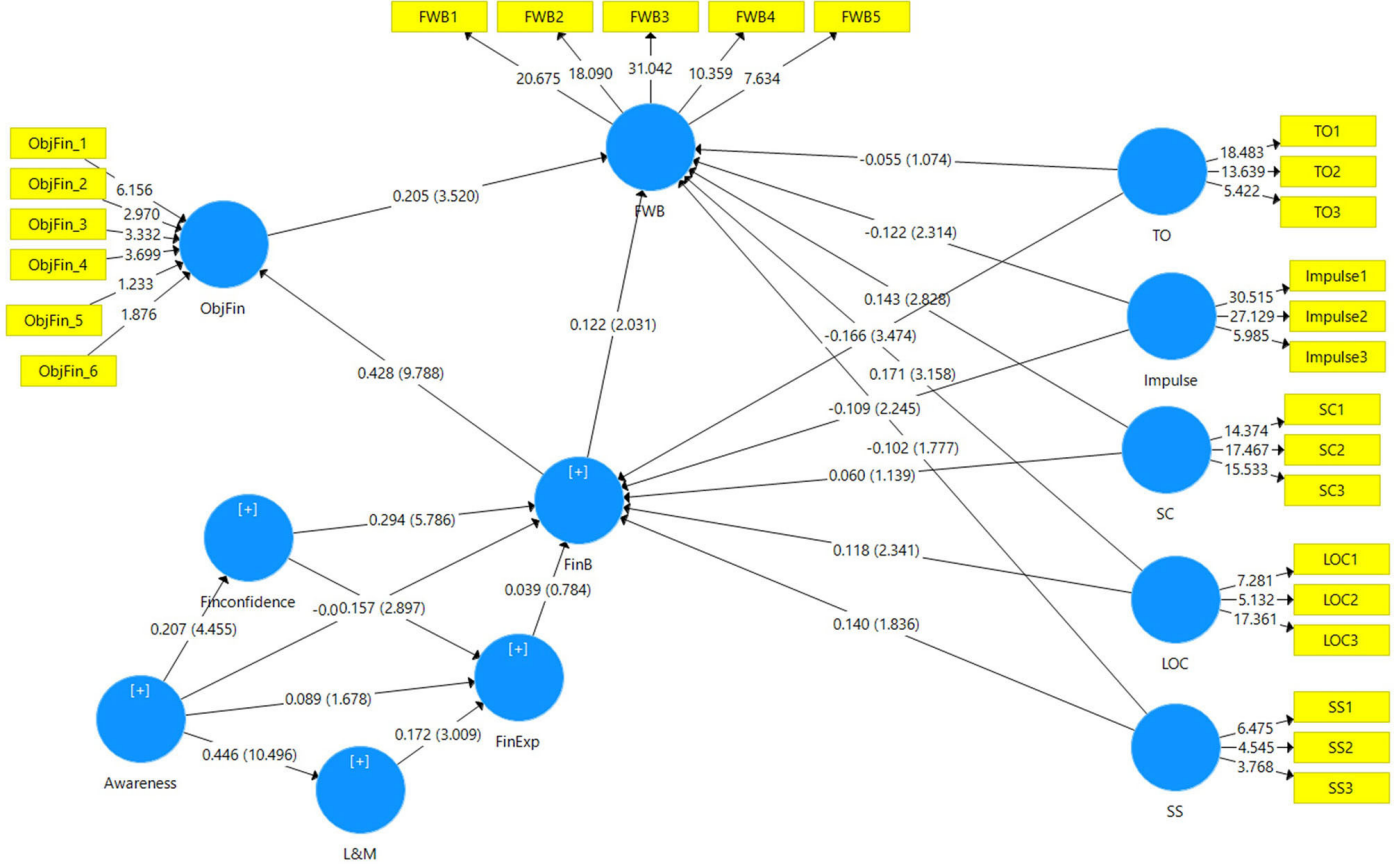
Relevance of the structural model paths. Source: SmartPLS output; In parenthesis is the *t*-values at 5,000 bootstraps.

Illustrative representation of the significance of the paths of the model based on significant beta values along with the *R*^2^ values of each endogenous construct is presented in Figure 3, and the relevance of the path models as established by the bootstrapping method is presented in Figure 4. It can be established that 17 out of the 21 direct path relations are significant, with only four insignificant direct relationships.

In complex models like ours, various endogenous constructs are being impacted in the model not only directly but also indirectly. Thus, for the complete evaluation and assessment of the structural model, the total effect (TE) of a specific exogenous construct on an endogenous construct needs to be considered. For instance, responsible FinB directly and significantly impacts FWB [FinB –> FWB] with β = 0.122, *t* = 2.034, *p* = 0.042 and also indirectly *via* objective financial situation [FinB –> ObjFin –> FWB] with β = 0.088, *t* = 3.301, *p* = 0.001 taking the total effect of FinB on FWB to 0.21, *t* = 3.718, *p* < 0. Table 8 displays the details of the total effects of each exogenous construct on each endogenous construct.

**Table 8:** Significance of total effects.

**Path**	**Original sample** ** (O)**	**Sample mean** ** (M)**	**Standard deviation** ** (STDEV)**	**T-statistics** ** (|O/STDEV|)**	***p*-values**	**Sig**.	**CI**	
							**2.5%**	**97.5%**
Awareness –> FWB	0.013	0.013	0.011	1.210	0.226	NS	−0.006	0.037
Awareness –> FinB	0.062	0.062	0.046	1.362	0.173	NS	−0.032	0.152
Awareness –> FinExp	0.198	0.197	0.044	4.471	0.000	***	0.114	0.284
Awareness –> Finconfidence	0.207	0.207	0.047	4.398	0.000	***	0.116	0.297
Awareness –> L&M	0.446	0.445	0.043	10.416	0.000	***	0.358	0.529
Awareness –> ObjFin	0.027	0.027	0.021	1.296	0.195	NS	−0.013	0.070
FinB –> FWB	0.210	0.206	0.056	3.711	0.000	***	0.094	0.314
FinB –> ObjFin	0.428	0.436	0.044	9.816	0.000	***	0.344	0.517
FinExp –> FWB	0.008	0.008	0.011	0.748	0.454	NS	−0.012	0.032
FinExp –> FinB	0.039	0.039	0.051	0.769	0.442	NS	−0.059	0.137
FinExp –> ObjFin	0.017	0.017	0.022	0.752	0.452	NS	−0.026	0.061
Finconfidence –> FWB	0.063	0.062	0.021	2.987	0.003	***	0.024	0.107
Finconfidence –> FinB	0.300	0.299	0.050	6.021	0.000	***	0.200	0.397
Finconfidence –> FinExp	0.157	0.156	0.054	2.911	0.004	***	0.053	0.263
Finconfidence –> ObjFin	0.128	0.131	0.026	4.879	0.000	***	0.081	0.185
Impulse –> FWB	−0.145	−0.145	0.050	2.890	0.004	***	−0.245	−0.047
Impulse –> FinB	−0.109	−0.110	0.048	2.260	0.024	**	−0.205	−0.013
Impulse –> ObjFin	−0.047	−0.048	0.022	2.136	0.033	**	−0.092	−0.005
L&M –> FWB	0.001	0.001	0.002	0.694	0.488	NS	−0.002	0.006
L&M –> FinB	0.007	0.007	0.010	0.704	0.481	NS	−0.010	0.028
L&M –> FinExp	0.172	0.171	0.057	3.009	0.003	***	0.057	0.282
L&M –> ObjFin	0.003	0.003	0.004	0.690	0.490	NS	−0.005	0.012
LOC –> FWB	0.196	0.200	0.052	3.798	0.000	***	0.096	0.300
LOC –> FinB	0.118	0.116	0.050	2.358	0.018	**	0.014	0.212
LOC –> ObjFin	0.051	0.051	0.023	2.213	0.027	**	0.006	0.096
ObjFin –> FWB	0.205	0.210	0.057	3.600	0.000	***	0.095	0.318
SC –> FWB	0.156	0.158	0.050	3.107	0.002	***	0.059	0.259
SC –> FinB	0.060	0.061	0.051	1.182	0.237	NS	−0.037	0.161
SC –> ObjFin	0.026	0.027	0.023	1.139	0.255	NS	−0.016	0.072
SS –> FWB	−0.072	−0.076	0.063	1.145	0.252	NS	−0.181	0.062
SS –> FinB	0.140	0.136	0.073	1.928	0.054	*	−0.047	0.249
SS –> ObjFin	0.060	0.059	0.033	1.835	0.067	*	−0.020	0.113
TO –> FWB	−0.090	−0.091	0.051	1.757	0.079	*	−0.187	0.012
TO –> FinB	−0.166	−0.169	0.048	3.496	0.000	***	−0.259	−0.072
TO –> ObjFin	−0.071	−0.074	0.022	3.227	0.001	***	−0.117	−0.030

*Source: SmartPLS output*.

*** *p <0.01*,

** *p <0.05*,

* *p <0.1, and NS, not significant*.

Out of 35 total effects, 23 are found to be significant with an adjusted R-squared value of 0.247 for FWB, 0.211 for FinB, and 0.181 for Objfin. These values of *R*^2^ are considered moderate and satisfactory (Cohen, 1988; Raithel et al., 2012; Ramalho and Forte, 2019). Further effect size (*f* ^2^) of awareness on objective financial literacy (L and M) is 0.249, and that of responsible FinB on ObjFin is 0.244 which is considered medium (Hair et al., 2017). Furthermore, all the constructs have a *Q*^2^ value of more than zero (i.e., *Q*^2^ > 0). The *Q*^2^ values FWB = 0.1, FinB = 0.18, FinExp = 0.08, Finconfidence = 0.04, L and M = 0.19, and ObjFin = 0.04 indicate that each endogenous construct of the model has predictive relevance (Fornell and Cha, 1994; Chin, 1998), and are calculated using Stone–Geisser criterion (Geisser, 1974; Stone, 1974) with an omission distance of 7.

The analysis in the preceding sections helps us conclude that out of 17 tested effects, 12 are observed to be statistically significant. Table 9 presents the summary of the hypothesized relations while giving the details of the estimate along with the t and the *p*-value.

**Table 9:** Hypothesis testing results.

**Hypothesis**		**Particulars**			**Result**
		**β**	***t*-value**	***p*-value**	
H1	Awareness –> L&M–> Financial expereince –> FinB	0.003	0.700	0.484	Not supported
H2_a_	Awareness–> Financial confidence–> FinB	0.061	3.407	0.001	Supported
H2_b_	Awareness–> Financial confidence–>Financial experience–> FinB	0.001	0.696	0.487	Not supported
H3	L&M–> Financial experience–> FinB	0.007	0.704	0.481	Not supported
H4_a_	Short-Term time orientation –> (–ve) FinB	−0.166	3.496	0.000	Supported
H4_b_	Impulsivity–> (–ve) FinB	−0.109	2.26	0.024	Supported
H4_c_	Self-Control–> FinB	0.06	1.182	0.237	Not supported
H4_d_	Locus of control–> FinB	0.118	2.358	0.018	Supported
H4_e_	Social status–> FinB	0.14	1.928	0.054	Supported
H5_a_	Short-Term time orientation –> (–ve) Perceived financial well-being	−0.056	1.079	0.079	Not supported
H5_b_	Impulsivity–> (–ve) Perceived financial well-being	−0.122	2.346	0.019	Supported
H5_c_	Self-Control–> Perceived financial well-being	0.143	2.811	0.005	Supported
H5_d_	Locus of control–> Perceived financial well-being	0.171	3.178	0.001	Supported
H5_e_	Social status–> (–ve) Perceived financial well-being	−0.102	1.751	0.080	Supported
H6	FinB–> Objective financial situation (ObjFin)	0.428	9.816	0.000	Supported
H7	FinB–> Perceived financial well-being	0.122	2.061	0.039	Supported
H8	Objective financial situation (ObjFin)–> Perceived financial well-being	0.205	3.600	0.000	Supported

*Source: Self compilation based on empirical results. L and M, objective financial knowledge; FinB, responsible financial behavior*.

## Results and Discussions

It can be concluded that 12 out of the 17 tested effects in the form of eight hypotheses, as proposed in the research model, are empirically supported. Specifically, we provide empirical evidence that responsible financial behaviors (FinBs), psychological factors, components of financial literacy, and objective financial situation have a significant effect on the financial well-being (FWB) of an individual. In addition to establishing these results, the reliability and validity of the constructs were within the threshold limits. Value of the coefficient of determination (*R*^2^) extracted for perceived FWB is 26%, and responsible FinB is 22.7%. These are considered as moderate values (Ramalho and Forte, 2019; Castro-González et al., 2020), especially in consumer behavior regarding finances. This value of variance is at par with or above those accounted for by other recent studies formulating complex FinB models by PLS-SEM in developing countries (Ali et al., 2015; Ramalho and Forte, 2019; Zulaihati et al., 2020).

Thus, we confirm the satisfactory level of the model by not just the value of *R*^2^ but also the effect size and predictive relevance. The empirical evidence confirms substantial to moderate effect size (*f* ^2^) of constructs of interest, namely, objective financial situation, responsible FinB, financial awareness, and psychological factors (time orientation and locus of control) (Cohen, 1988). Further, the *Q*^2^-values of all the endogenous constructs with an omission distance of seven have a non-negative and above zero value, establishing the predictive relevance of the model.

Among all the factors directly associated with responsible financial behavior (FinB) in the complete model, financial confidence (β = 0.294, *t* = 5.787, *p* < 0.001) has the highest effect followed by psychological factors time orientation with a high negative impact (β = −0.166, *t* = 3.496, *p* < 0.001) and social status with a positive impact (β = 0.14, *t* = 1.92, *p* = 0.05). The strength of these relationships does not observe much difference when indirect effects are accounted for, and the only increase in the β values that is observed is that for financial confidence (β = 0.3, *t* = 6.021, *p* < 0.001). The results imply that individuals with higher confidence in their financial skills have higher responsible FinBs. This observation is consistent with the results of Bannier and Neubert (2016), Farrell et al. (2016), and Fernandes et al. (2014), those asserted that greater confidence is linked with responsible FinBs, such as higher investments, savings, the likelihood of retirement planning, and better credit score.

Allgood and Walstad (2016) found the confidence level to have a more substantial influence on the financial behaviors (FinBs) as compared with financial knowledge. Further, based on the meta-analysis of 201 prior studies, a weak relationship between financial literacy and FinBs, with financial literacy merely explaining 0.1% of the variation in FinBs, is observed by Fernandes et al. (2014). A similar result is obtained for the conceptual model in which confidence has emerged as a superior predictor of responsible FinB in contrast to other components of financial literacy, such as objective financial knowledge. The result that factual or objective knowledge is not a sufficient driver of FinBs finds support in the studies of Kiviat and Morduch (2012) and Serido et al. (2013).

While analyzing the significant effects of various psychological factors impacting responsible financial behavior, it is observed that high social status has (β = 0.14, *t* = 1.92, *p* = 0.05) the highest positive effect followed by an internal locus of control (β = 0.118, *t* = 2.358, *p* = 0.018). The results imply that individuals with high concerns for society, i.e., high social status score and with an internal locus of control, i.e., feeling in control of their destiny, are involved in responsible FinBs. The positive relationship of social status with FinBs is also established by Frederiks et al. (2015), Kempson et al. (2013), and Sundarasen et al. (2016). These findings are also consistent with previous studies that have reported that internal locus of control is linked with positive FinBs, such as high savings rates (Cobb-Clark et al., 2016), better personal financial management (Perry and Morris, 2005; Angulo-Ruiz and Pergelova, 2015; Mien and Thao, 2015), and informed product choice (Hoffman et al., 2003).

Inverse relationship is observed for time orientation (β = −0.166, *t* = 3.496, *p* < 0.001) and impulsivity (β = −0.109, *t* = 2.26, *p* = 0.024) with responsible FinB. These results imply that individuals with short-term time orientation and high impulsivity show poor FinB. These results are consistent with observations where long-term time orientation is associated with retirement savings (Jacobs-Lawson and Hershey, 2005), less credit (Benton et al., 2007), and higher savings (Howlett et al., 2008). Further, impulsivity is associated with lower retirement savings (DeHart et al., 2016), over-indebtedness, and lower savings (Gathergood and Weber, 2014).

Thus, from the above discussion, we can infer that individuals with higher confidence in their skills to take financial decisions, long-term time orientation, more concern for social status, low impulsivity, and high locus of control are expected to engage in responsible FinB. At the lower order of the construct, savings (β = 0.244, *t* = 33.912), credit aversion (β = 0.238, *t* = 30.448), and spending restraint (β = 0.236, *t* = 31.33) emerge as the most significant and positive relations for responsible FinB. These results suggest that among all the responsible financial behaviors evaluated, active savings, credit management, and spending restraint are the most critical ones.

For objective financial situation, responsible FinB (β = 0.428, *t* = 9.816, *p* < 0.001) has the highest significant positive impact followed by financial confidence (β = 0.128, *t* = 4.879, *p* < 0.001). This result implies a better objective financial situation for individuals with high financial confidence and responsible FinB. Among the psychological factors, LOC and SS have a significant positive relationship with the objective financial situation. These relationships imply that individuals with an internal locus of control and a high concern for social status have a better objective financial situation. Extant literature supports a positive association of internal locus of control with objective financial situations, such as higher earnings (Heineck and Anger, 2010), wealth accumulation (Cobb-Clark et al., 2016), and financial status (Morgan and Eckert, 2004). Further, impulsivity and time orientation have a significant negative relationship with the objective financial situation. This relationship implies that high impulsivity and short-term time orientation are associated with the poor objective financial situation.

Among all the factors affecting FWB, the direct effect of the objective financial situation (β = 0.205, *t*=3.6, *p* < 0.001) and locus of control (β = 0.171, *t* = 3.178, *p* < 0.001) is observed to be highest. Positive and significant association of objective financial situation with financial satisfaction, which can be considered a proxy of FWB, is also reported by Shim et al. (2009) and Xiao et al. (2014). The strength of the relationships of various constructs changes when considering the total-effects they have on the construct of interest, i.e., perceived FWB. The strength of the relationship of responsible FinB with FWB increases from 0.122 to 0.21 when considering the total effect. Responsible FinBs positively impact the objective financial situation, which in turn positively and significantly impacts the perceived FWB of an individual (β= 0.21, *t* = 3.711, *p* < 0.001), as also evident in the results of Rowley et al. (2012) that adopting responsible FinBs facilitate individuals to take better financial decisions and also helps them cope with changes. A positive direct link of responsible FinB and FWB is also established in the studies of Kempson et al. (2013), Kempson et al. (2017) and Netemeyer et al. (2018).

Among the psychological factors impacting financial well-being (FWB) perception, internal locus of control (β = 0.196, *t* = 3.798, *p* < 0.001) has the highest positive impact, consistent with observations of Kempson et al. (2017), Mahdzan et al. (2019), and Prawitz and Cohart (2016). This is followed by self-control (β = 0.156, *t* = 3.107, *p* = 0.002), consistent with Strömbäck et al. (2017). Impulsivity (β = −0.145, *t* = 2.89, *p* = 0.004), and social status (β = −0.102, *t* = 1.751, *p*= 0.08) have significant negative effect on FWB. These results imply that individuals with a high internal locus of control, i.e., they feel they are in control of their destiny, and self-control have higher perceived FWB. On the other hand, individuals with high impulsivity and high concern for society have a negative perception about their FWB, as is evident in the studies of Kempson and Poppe (2018), Shepard and Turner (2019), and Strömbäck et al. (2017).

Surprisingly, none of the components of financial literacy other than financial confidence (β = 0.063, *t* = 2.987, *p* = 0.003) has a significant positive relationship with perceived FWB. The empirical results also provide strong evidence to support the relationship between the constituents of financial literacy (awareness and confidence) with responsible FinB, i.e., Awareness –> Finconfidence –> FinB. This implies higher financial awareness results in higher financial confidence, which, in turn, influences the FinBs positively. These results are consistent with the observation by Allgood and Walstad (2016), which reported that financial confidence is the better predictor of FinB as compared with financial knowledge. It can be argued that as confidence and perception are both self-felt phenomena, they need not be associated with the underlying objective knowledge and are more likely to move in the same direction, whereas the other constituents of financial literacy, i.e., objective financial knowledge and financial experience, were more factual. Thus, there is a possibility that an individual may have lower levels of objective financial knowledge while having a higher confidence level.

Empirical results provide strong evidence of the relationship between responsible FinB, objective financial situation and perceived financial well-being (FWB), i.e., FinB –> ObjFin–> FWB. This result implies that individuals who engage in responsible FinB experience a higher objective financial situation, which ultimately leads to a higher perception of FWB. Responsible FinB encompasses a basket of positive financial management behaviors, such as saving regularly, not overspending, making informed choices, etc. The positive relationship between responsible FinB and objective financial situation indicates that individuals who report engaging in or believing in traits of responsible FinBs can achieve a higher objective financial situation. For example, individuals who believe in saving regularly (responsible FinB) report higher levels of savings in terms of income saved (objective financial situation).

Further, the strong positive relationship between objective financial situation and perceived FWB implies that individuals experience higher FWB when they have a high objective financial situation. This relationship implies that the perception of being financially well is supported by the real objective financial situation of an individual (i.e., savings level, loan due, contingency planning, etc.). Thus, the objective financial situation goes hand in hand with the perception of one's FWB. These results find support in the studies of Shim et al. (2009) and Xiao et al. (2014) that reported good FinB leads to higher subjective FWB, while irresponsible FinB adversely affects the FWB of an individual (Kim and Garman, 2003).

## Conclusion

This research study presented results of an empirically tested holistic financial well-being (FWB) model in a developing economy context. The results showed that the objective financial situation of an individual, as measured by self-reported ease of meeting routine financial commitments, level of liquid savings, status of credit due, ability to absorb financial shock, diversification of investment portfolio, and retirement planning, is positively correlated with her/his perceived/subjective FWB (β = 0.205, *p* < 0.001). However, FWB and objective financial situation are not perfect correlates. This result implies that the subjective FWB of individuals, as captured in the study, not only reflects the well-being of individuals as measured by the traditional objective wellness indicators, but it also reflects other possible contributors of FWB, such as experiences and expectations of individuals.

Financial behavior is associated with financial well-being directly and indirectly *via* objective financial situation. Although both the relationships are significant, a higher direct positive relationship is observed between FinBs and the objective financial situation (β = 0.428, *p* < 0.001). This implies that higher responsible FinB (as gauged by credit aversion, informed product choice, active saving, spending restraint, informed decision-making, planning, monitoring, and daily ease) is associated with a better objective financial situation, which, in turn, is related to higher perceived FWB. However, there are still certain aspects of responsible FinB that are not reflected *via* objective financial situation, but they directly enhance perceived FWB (β = 0.122, *t* = 2.061, *p* = 0.039). While this finding is not surprising, it reinforces the existing knowledge of the vital link between responsible FinB and the objective financial situation, i.e., what we do/actions and the results. This result has an important implication for the overall well-being and FWB, specifically.

The empirical evidence provides that psychological factors, namely, high impulsivity and concern for social status have significant negative relation, whereas internal locus of control and self-control have a significant positive relationship with FWB. Further, internal locus of control emerged as the highest impacting factor followed by self-control. High impulsivity and short-term time orientation are inversely related to responsible FinB, whereas internal locus of control and social status are observed to impact responsible FinB positively. Furthermore, short-term time orientation emerges to be the highest impacting factor, followed by social status.

The financial literacy components that significantly influenced responsible behavior are financial confidence and financial awareness. These results support the previous literature that contends limited role of objective financial knowledge in explaining FinBs when compared with factors related to personal characteristics, such as confidence and other psychological traits.

The strength of this study can be concluded in its contribution to the literature by introducing multiple personal, financial literacy, and behavioral components in a single framework and subsequently applying them to a novel context, i.e., South Asian developing country (India). Furthermore, FWB is measured using both subjective and objective measures. Although a sincere attempt is made to analyze and report FWB in a holistic manner, the respondents of this study are from urban areas, leaving scope for further research on rural population or comparative research that could test whether geography plays a role in determining FinBs and FWB. Further, the objective financial situation score in this study is derived from the self-reported data of the respondents. Banks or financial institutions, in the capacity of having access to personal financial data, can conduct research that further enhances the understanding of the relationships by comparing the reported and actual data on the financial situation.

This study is an earnest attempt to contribute to the growing body of scientific literature in the field. However, there remains a scope for further improvement. This study is based on respondents from urban areas, and further research can be conducted on rural population. Also, comparative research that could test whether geography plays a role in determining FinBs and FWB can be conducted. Thus, the results of the study should be interpreted with caution before generalizing them, as they are based on a specific sample without conducting a power analysis for the model.

## Data Availability Statement

The raw data (without respondents personal details) will be made available for academic use with due credit given to the authors.

## Author Contributions

KS was involved in all the steps of the process and was the primary writer of the text. GT was involved in the questionnaire development, data collection and analysis. MV supervised in the research design and analysis as well as write up of the text. All authors contributed to the article and approved the submitted version.

## Conflict of Interest

The authors declare that the research was conducted in the absence of any commercial or financial relationships that could be construed as a potential conflict of interest.
