# Supplementary material for: Understanding the Path Toward Financial Well-Being: Evidence From India
Source: Front Psychol. 2021 Jul 21;12:638408. doi: 10.3389/fpsyg.2021.638408 (PMC8335566; doi:10.3389/fpsyg.2021.638408)

**Appendix A: Discriminant Validity Reflective Measurement Model**

**Table A1: Fornell-Larcker Criterion**

Source: Survey results; SIC= Single item construct

**Table A2: HTMT Criterion**

Source: Survey results

**Appendix B: Discriminant Validity Formative Measurement Model**

**Table B1: Fornell-Larcker Criterion**


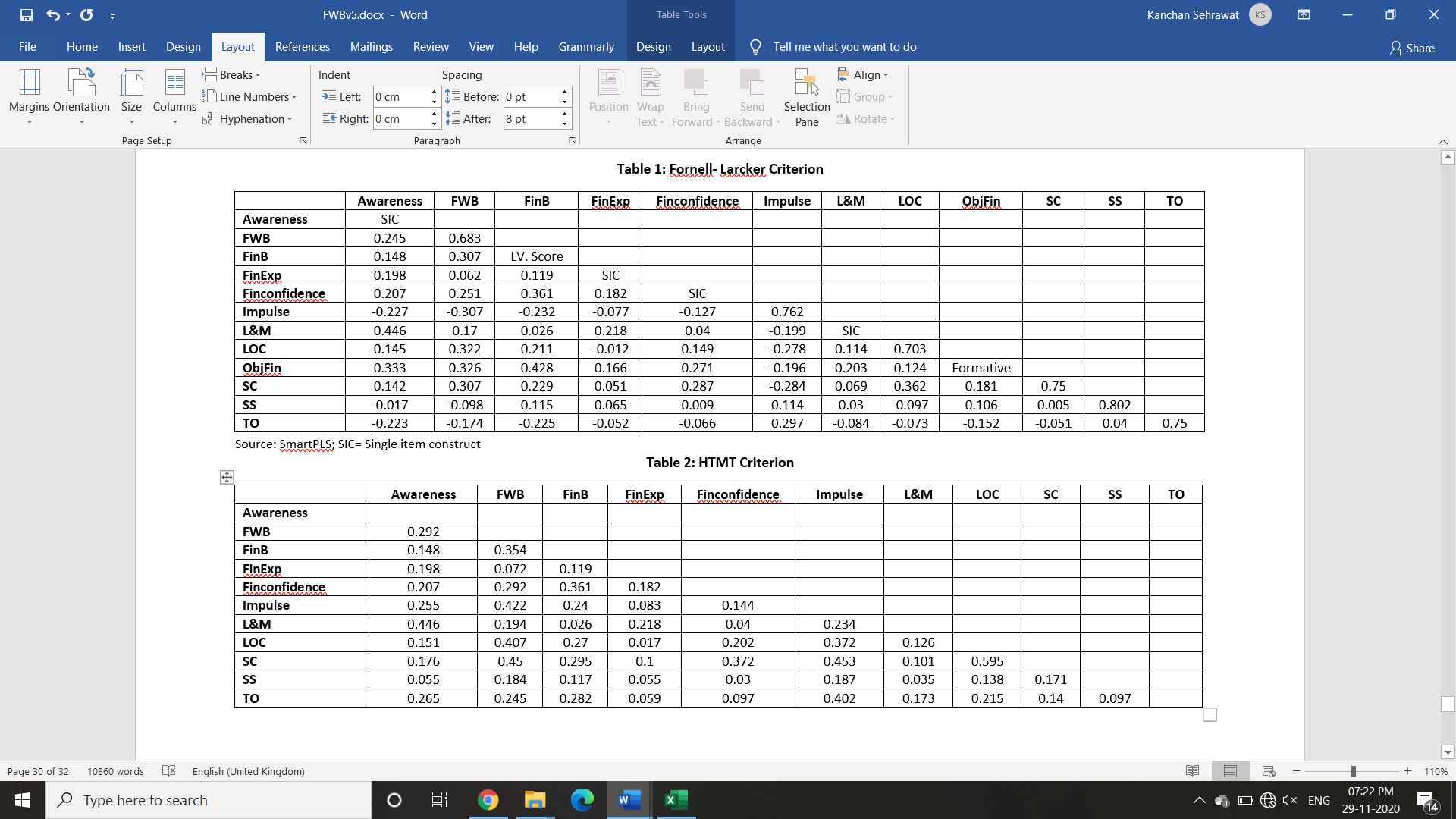


**Table B2: HTMT Criterion**


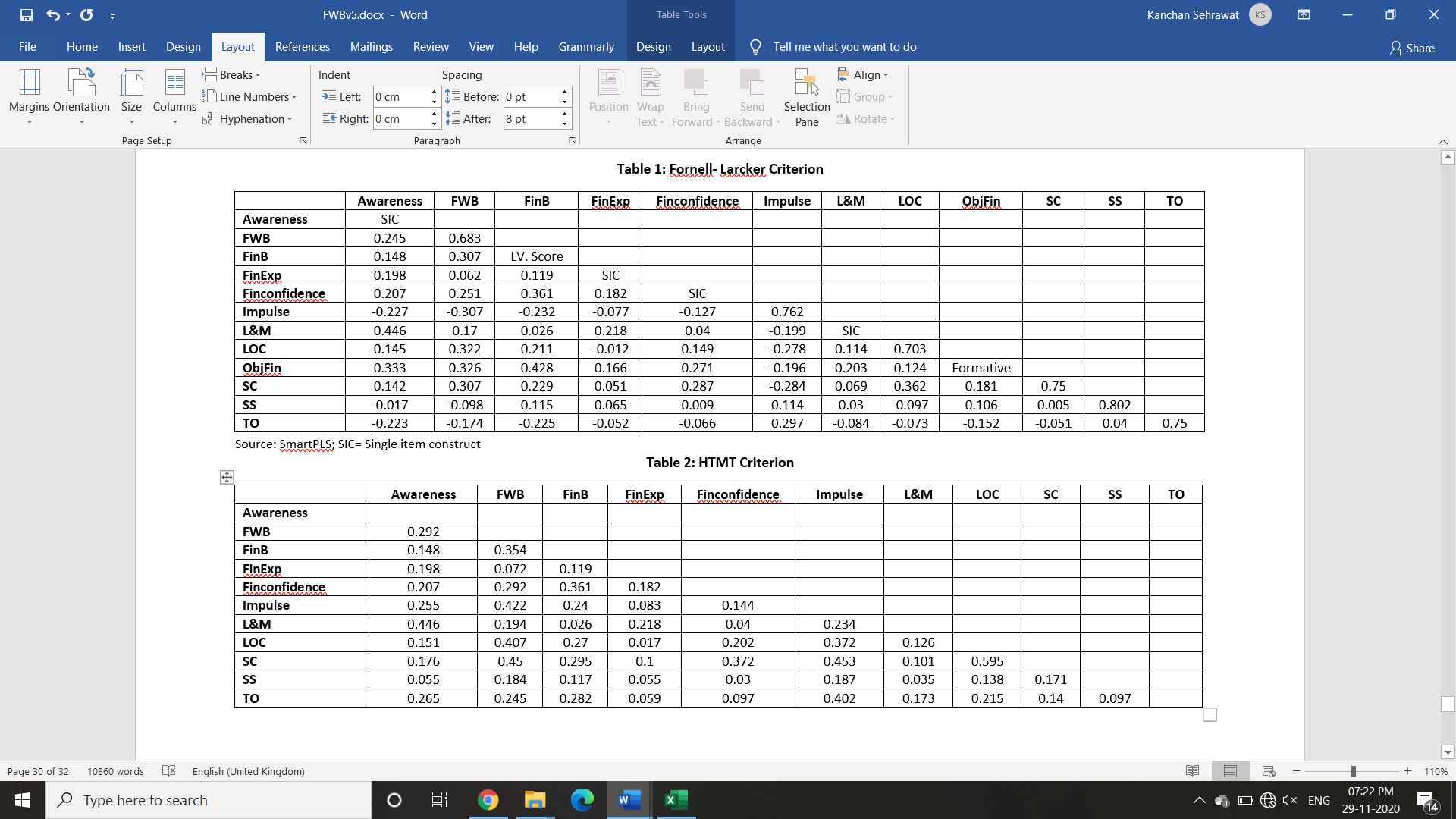

Supplement: Supplementary file 1 [file Table_1.DOCX]
